# Effect of short-term hindlimb immobilization on skeletal muscle atrophy and the transcriptome in a low compared with high responder to endurance training model

**DOI:** 10.1371/journal.pone.0261723

**Published:** 2022-01-13

**Authors:** Jamie-Lee M. Thompson, Daniel W. D. West, Thomas M. Doering, Boris P. Budiono, Sarah J. Lessard, Lauren G. Koch, Steven L. Britton, Nuala M. Byrne, Matthew A. Brown, Kevin J. Ashton, Vernon G. Coffey

**Affiliations:** 1 Faculty of Health Sciences and Medicine, Bond University, Robina, Australia; 2 Department of Physiology and Membrane Biology, University of California, Davis, Davis, California, United States of America; 3 Toronto Rehabilitation Institute, Toronto, Canada; 4 Faculty of Kinesiology and Physical Education, University of Toronto, Toronto, Canada; 5 School of Health, Medical and Applied Sciences, Central Queensland University, Rockhampton, Australia; 6 School of Community Health, Charles Sturt University, Bathurst, Australia; 7 Research Division, Joslin Diabetes Center, Boston, Massachusetts, United States of America; 8 Department of Medicine, Brigham and Women’s Hospital and Harvard Medical School, Boston, Massachusetts, United States of America; 9 Department of Physiology and Pharmacology, The University of Toledo College of Medicine and Life Sciences, Toledo, Ohio, United States of America; 10 Department of Anesthesiology, University of Michigan, Ann Arbor, Michigan, United States of America; 11 School of Health Sciences, University of Tasmania, Hobart, Australia; 12 National Institute for Health Research, Guy’s and St Thomas’ Biomedical Research Centre, Kings College London, London, United Kingdom; Cinvestav-IPN, MEXICO

## Abstract

Skeletal muscle atrophy is a physiological response to disuse, aging, and disease. We compared changes in muscle mass and the transcriptome profile after short-term immobilization in a divergent model of high and low responders to endurance training to identify biological processes associated with the early atrophy response. Female rats selectively bred for high response to endurance training (HRT) and low response to endurance training (LRT; n = 6/group; generation 19) underwent 3 day hindlimb cast immobilization to compare atrophy of plantaris and soleus muscles with line-matched controls (n = 6/group). RNA sequencing was utilized to identify Gene Ontology Biological Processes with differential gene set enrichment. Aerobic training performed prior to the intervention showed HRT improved running distance (+60.6 ± 29.6%), while LRT were unchanged (-0.3 ± 13.3%). Soleus atrophy was greater in LRT vs. HRT (-9.0 ±8.8 vs. 6.2 ±8.2%; P<0.05) and there was a similar trend in plantaris (-16.4 ±5.6% vs. -8.5 ±7.4%; P = 0.064). A total of 140 and 118 biological processes were differentially enriched in plantaris and soleus muscles, respectively. Soleus muscle exhibited divergent LRT and HRT responses in processes including autophagy and immune response. In plantaris, processes associated with protein ubiquitination, as well as the atrogenes (*Trim63* and *Fbxo32*), were more positively enriched in LRT. Overall, LRT demonstrate exacerbated atrophy compared to HRT, associated with differential gene enrichments of biological processes. This indicates that genetic factors that result in divergent adaptations to endurance exercise, may also regulate biological processes associated with short-term muscle unloading.

## Introduction

Skeletal muscle has a high degree of plasticity, quickly adapting its structural and functional properties in response to disruption of homeostasis. The adaptive response and ensuing beneficial effects of endurance exercise on skeletal muscle metabolism are well known. However, the magnitude of response to a standard bout of contractile activity shows substantial variation between individuals and the contribution of genetic, heritable factors in determining the individual adaptive response is a topic of ongoing scientific scrutiny [1–3]. To account for heterogeneity and better understand genetic factors in the response to training, a selective breeding model has been developed that demonstrates improved running capacity and metabolic function in high responders to endurance training (HRT), compared with low responders to endurance training (LRT) that have reduced running capacity and impaired metabolism [4].

Chronic diseases such as cancer, diabetes and obesity, and the effects of aging leading to sarcopenia, are often accompanied by shared maladaptation responses in skeletal muscle [5]. Muscle atrophy is a secondary complication in many diseases and with injury where bedrest and/or immobilization reduces physical activity and promotes loss of muscle mass. Studies reporting individual responses to muscle unloading are limited but there is evidence indicating significant heterogeneity in muscle atrophy following limb immobilization [6, 7]. As such, experimental models of contrasting adaptive responses may provide new information on genetic contributions and biological processes with maladaptation such as muscle atrophy. Moreover, whether the response to exercise training is also associated with the severity of immobilization-induced muscle atrophy is currently unknown.

Divergent metabolic and functional capacity between HRT and LRT indicates that LRT may be genetically predisposed for muscle loss compared with HRT. Poor metabolism and dysregulated molecular signalling in LRT [4] may contribute to a cellular milieu that dysregulates processes important in the control of muscle mass and remodelling. Indeed, we have previously shown that LRT have an attenuated compensatory hypertrophy response to functional overload induced via surgical ablation of synergist muscles [8]. Consequently, we reasoned that the same heritable factors that induce low response to aerobic training may also alter the mechanisms controlling atrophy. Here, we examined changes in skeletal muscle mass and the enrichment of gene sets, identified via RNA sequencing (RNAseq), in the early response to hindlimb immobilization in HRT and LRT. We hypothesized that LRT would exhibit greater atrophy compared with HRT, and that divergent responses would also be evident in distinct gene set enrichment maps of biological processes.

## Materials and methods

Rats were obtained from a bi-directional selective breeding program that has been described in detail previously [9]. Briefly, genetically heterogeneous rats from the highest and lowest 10^th^ percentile for endurance adaptations to an eight-week treadmill training program were selected as breeders for each subsequent generation using a rotational breeding system that reduced the rate of inbreeding relative to random breeding. At each generation ~100 rats per line were phenotyped for their training response to the treadmill training program. Endurance adaptation was defined as post-training exercise capacity minus pre- training exercise capacity. Total running distance, work performed, and time-to-fatigue variables during treadmill tests were recorded and calculated as previously described [9]. Training began at ∼12 weeks of age and exercise sessions were undertaken three days per week (total 24 training sessions). The training protocol provided a total of 618 minutes (>10 hours) of running time, a total distance of ∼9.9 km, and a cumulative vertical gain of ∼2.5 km. Twenty-four female rats (12 HRT and 12 LRT) from the 19th generation were used for this study due to larger training responses than males and to extend on previous work using the LRT/HRT model [9]. Rats were housed in temperature- and humidity-controlled facilities on a 14:10 hour light-dark cycle with ad libitum access to standard chow (20% protein, 4.8% fat) and water. Rats began the 3 day experimental period at 14 months of age to maximize the latent period after treadmill training before the hindlimb immobilization because the extent to which phenotype changes with endurance training such as mitochondrial and oxidative metabolism adaptation might remain evident in HRT rats is unknown. Rats were randomly assigned to either an immobilization group or control group (n = 6/group). All experimental procedures undertaken during the study were approved by the University Committee of Use and Care of Animals at the University of Michigan and Queensland University of Technology Animal Ethics Committee (1300000531).

### Hindlimb immobilization and muscle collection

Rats were exposed to anaesthesia with 2–4% isoflurane inhalation in an individual chamber followed by nose cone inhalation that was maintained throughout all surgical procedures in the study [8]. Rats underwent hindlimb immobilization via casting to induce atrophy of the hindlimb muscles. Briefly, one hindlimb was shaved and wrapped with fiberglass casting tape (3M VetCast Plus veterinary casting tape). The foot was held in plantar-flexion while the casting tape hardened to facilitate muscle atrophy. In the 3 day intervention period prior to muscle collection, animals were monitored daily for signs of discomfort or pain; none of the animals showed any signs of undue stress with no observable weight bearing of the immobilized limb apparent during twice daily observation.

After 3 days of immobilization, rats received puromycin injections (0.02 μmol/g body wt, i.p.) 30 minutes prior to muscle collection to measure muscle protein synthesis as previously described [10–12]. Thereafter, plantaris and soleus muscles from the immobilized limb were excised, weighed and snap-frozen in liquid nitrogen for further analyses [8]. Rats from the LRT and HRT control groups (no hindlimb immobilization) were also anaesthetized and the plantaris and soleus muscles were removed using the same procedures for comparison on the same day. Rats were terminated after removal of plantaris muscles at the conclusion of the experimental period under general anaesthesia by permanent cessation of circulation (Annex IV in the European Directive 2010/63/EU). We employed separate control groups for analysis to mitigate potential confounding effects of a contralateral design on muscle overload, where greater reliance on the control limb for ambulatory cage activity might be evident with a hindlimb cast immobilization model [13].

### RNA extraction, library preparation, RNAseq and bioinformatics analysis

Total RNA from plantaris and soleus muscle was isolated using the miRNeasy mini kit (QIAGEN, Hilden, Germany) according to the manufacturer’s protocol. Briefly, ~50–80 mg tissue was homogenised in QIAzol with 0.9–2.0 mm RNase-free steel beads in a Bullet Blender Gold at 4°C (Next Advance, Troy, New York, United States) [8]. Total RNA was then further purified using RNeasy spin columns. RNA yield was determined using a Qubit 3.0 fluorometer (Thermo Fisher Scientific, Waltham, Massachusetts, United States), and RNA integrity was assessed using a 2100 Bioanalyzer (Agilent, Santa Clara, California, United States). RNA integrity scores were >8.0 for each sample. RNA sequencing was performed at the Australian Translational Genomics Centre (Queensland University of Technology, Australia) according to standard protocols [8]. Briefly, 1 μg of each RNA sample was used for library construction using the Illumina TruSeq Stranded Total RNA Library kit with Ribo-Zero Gold depletion (Illumina, San Diego, California, United States), as per the manufacturer’s instructions. Adapter-ligated fragments were amplified by PCR for 11 cycles. The quality and size of the final library preparations were analysed on a TapeStation (Agilent). Indexed samples were pooled and then sequenced on a NextSeq 500 system (Illumina), generating approximately 50 million paired-end 2 x 100-bp reads for each sample.

Bioinformatic analysis was carried out as previously described [8]. Briefly, length normalization scaled to TPM, transcript quantification, and quasi-mapping against the rat reference genome (Ensembl Rnor_6.0 release 91; cDNA and ncRNA) was performed using Salmon 0.9.1 [14]. Transcript reads were then imported into R/Bioconductor and summarised at the gene level using the tximport package [15]. Voom transformation, differential expression analysis and descriptive statistics were performed in limma [16, 17]. The linear model also incorporated RNA integrity as a covariate. The following pairwise comparisons were investigated: LRT hindlimb immobilization vs LRT control (HIinLRT), HRT hindlimb immobilization vs HRT control (HIinHRT) and the difference in atrophy with hindlimb immobilization (HIinLRT vs HIinHRT; DELTA). A false discovery rate (FDR) was applied to correct for multiple comparisons, with statistical significance accepted at FDR <0.001. Gene Set Enrichment Analysis (GSEA) was used to detect coordinated changes in gene expression of functionally related sets of genes. Gene set enrichments were analysed using the clusterProfiler package (10,000 permutations; gene set size range 25–500) and visualised as networks in Cytoscape using the EnrichmentMap package [18, 19]. Conservative threshold parameters were used; specifically, FDR <0.05, nominal P-value <0.001 and a combined similarity cut-off >0.375. Network clusters were further summarised and annotated using the AutoAnnotate package, with additional manual editing [20]. Genes were visualised in Cytoscape using the geneMANIA package as previously described [8, 21].

### RT-qPCR

Two-step reverse transcription quantitative PCR (RT-qPCR) with SYBR Green I was used to confirm differential gene expression for select transcripts (primer sequences shown in S7 Table in S1 File). Briefly, 400ng of total RNA was used to synthesize cDNA using the iScript cDNA Synthesis kit (Bio-Rad, Hercules, CA, USA) according to manufacturer’s protocol. Each RT-qPCR 10μL reaction contained 1x SYBR Green Supermix (Bio-Rad), 100nM of each primer and 4μL of a 1:40 dilution of cDNA and was assayed on a CFX96 qPCR system (Bio-Rad). Optimal qPCR cycling conditions consisted of an initial denaturation at 95°C for 3 min followed by 40 cycles of 95°C for 15 seconds and 62°C for 60 seconds. After the final PCR cycle, reactions underwent melt curve analysis to detect nonspecific amplicons. Reactions were performed in triplicate and expression levels were normalized to beta-2-microglobulin (*B2m*) as a reference gene. Several reference genes were tested including *B2m*, *Pgk1*, *Actb*, *Ppia*, and *Rpl13a*, with *B2m* being the most stable (M = 0.99). Changes in expression were calculated using the 2^-ΔΔCt^ method and statistically analysed in Prism (GraphPad software, La Jolla California, USA) using a one-way ANOVA, with significance accepted for P < 0.05.

### Western blotting

The methods employed for Western blotting have been provided in detail previously [22]. Briefly, skeletal muscle tissue (~40 mg) was stabilized in buffer with protease and phosphatase inhibitor cocktail (Bimake, USA) and homogenized twice for 4 min (10,000 Bullet Blender 24 Gold, Next Advance, USA). Protein content of lysate were quantified (Pierce BCA Protein Assay Kit, Thermo Scientific, USA) samples were equilibrated in 4 × Laemmli sample buffer with DTT and heated to 95°C for 10-min with 50 μg protein subsequently loaded into separate wells on acrylamide gels (Bio-Rad, USA) for electrophoresis and wet-transferred (120-min, 70 V; Polyvinylidene difluoride membrane, Bio-Rad, USA). Membranes were washed (3 × 5-min) in Tris-buffered saline with Tween (0.05%, TBST), then blocked at room temperature for 60 min in TBST and 5% skim milk powder. Membranes were incubated in puromycin primary antibody at 4°C overnight (1: 1000 MABE343, Merck Millipore, MA, USA) and then washed and incubated in secondary antibody. Chemiluminescent solution (SuperSignal West Femto Maximum Sensitivity Substrate, Thermo Fisher Scientific Inc., USA) was used to quantify blots by densitometry (ChemiDoc, Bio-Rad, USA) and quantified relative to total protein abundance (Amido Black Staining Solution 2 ×, Sigma-Aldrich, USA).

### Statistics

Two-way analysis of variance with Sidak’s multiple comparisons test was used to analyse endurance capacity, body mass, loss of plantaris and soleus muscle mass, muscle protein synthesis, and myosin heavy chain expression. Percent changes were analysed by unpaired t tests. Statistical analyses were performed in GraphPad Prism 7.03 (GraphPad software, CA USA). All data are presented as mean ± standard deviation (SD) and the level of statistical significance was set at P <0.05. Cohens d statistics were calculated to provide effect size for the magnitude of changes in skeletal muscle mass and muscle protein synthesis, and interpreted using accepted threshold values: 0.2, small; 0.5, moderate; and 0.8, large effects [23].

## Results

### Endurance capacity

**Fig 1:**
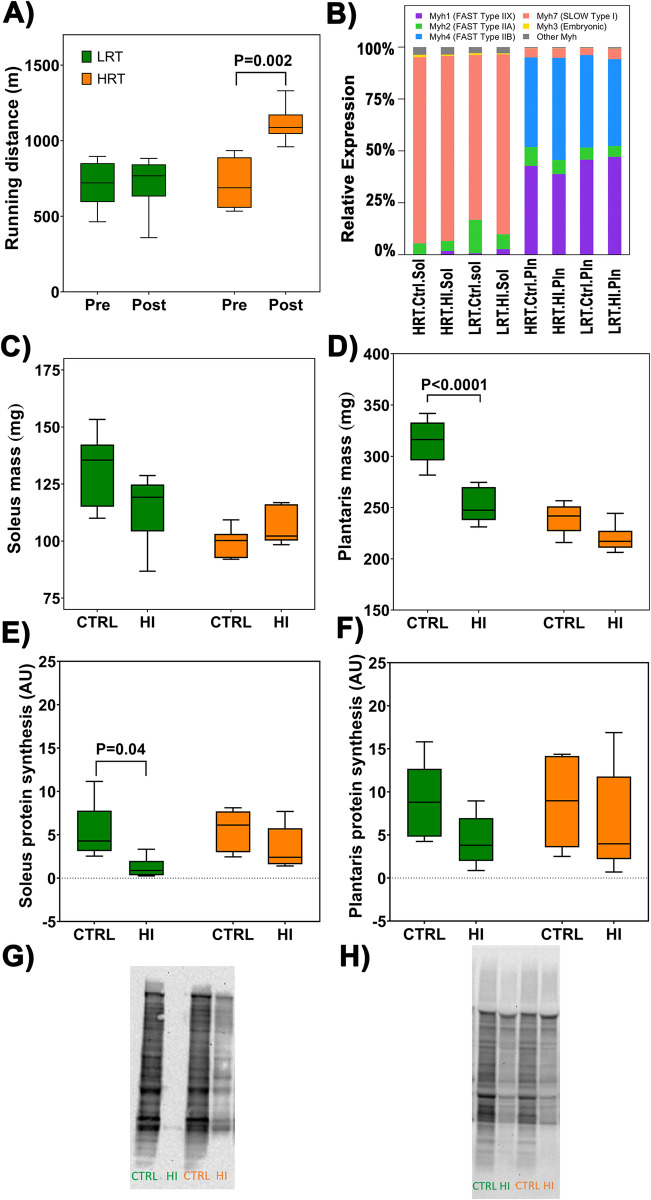
Phenotype data showing (**A**) running distance after an 8-wk treadmill training program, (**B**) myosin heavy chain (*Myh*) transcript expression, (**C**) soleus muscle mass and (**D**) plantaris muscle mass, (**E**) soleus muscle protein synthesis and (**F**) plantaris muscle protein synthesis, in hindlimb immobilization (3 d; HI) and control (CTRL) groups of selectively bred low- and high responders to endurance training (LRT vs. HRT). Boxes indicate the interquartile range (25%-75%) with the horizontal bar within each box indicating the median. The whiskers show the minimum and maximum values. P-values vs. breeding line-matched control (n = 6/group).

The response to endurance training was significantly different between LRT and HRT (P<0.0001) with HRT but not LRT increasing distance run after training (HRT baseline 714 ± 139 m vs. post-training 1110 ± 154 m, P<0.0001; Fig 1A). HRT also showed greater improvements than LRT in pre-to-post training work performed (77.7 ± 57.5%) and time-to-fatigue (36.1 ± 25.8%; both P<0.001 versus LRT).

### Myosin heavy chain gene expression

Myosin heavy chain (*Myh*) expression was examined to analyse muscle fibre phenotypes as well as validate RNAseq workflow by examining *Myh* expression in well-characterized muscle fibre phenotypes. Phenotypic analysis characterized myosin heavy chain (*Myh*) expression via transcriptomic data. Of the myosin heavy chain genes, *Myh1*, *Myh2*, *Myh4*, *Myh6*, and *Myh7* were most highly expressed (Fig 1B). As expected, there were clear differences in *Myh* between tissue type. In soleus tissue the most predominant transcript was *Myh7* (HRT 89.6%, LRT 79.4%), which encodes for the slow-twitch, myosin heavy chain-beta isoform (Type I fibres transcripts; S11 Table in S1 File). The most predominant transcripts in plantaris tissue were *Myh1* (HRT 42.8%; LRT 45.8%) and *Myh4* (HRT 43.2%; LRT 44.6%; Type II fibre transcripts), with lesser *Myh2* (HRT 9.0%; LRT 5.7%) and *Myh7* (HRT 4.5%, LRT 3.4%) expression (S11 Table in S1 File). Myosin transcripts in plantaris tissue were similar between groups and intervention.

### Body mass, skeletal muscle mass, and muscle protein synthesis

LRT body mass was ~20% greater than HRT at the time of endurance training. LRT and HRT decreased body mass after endurance training (-39 g and -36 g, respectively; both P<0.001), however body mass loss was not different between groups. There was also no significant difference in body mass between control and intervention groups at the time of the experimental period (LRT control 280 ± 41 vs HIinLRT 268 ± 23 g; HRT control 251 ± 19 vs HIinHRT 253 ± 28 g).

Soleus muscle mass decreased in LRT (-12.0 ± 12.7% [-15.7 ± 16.7 mg], *d* = -1.0, P = 0.10) and was slightly higher in HRT (+6.85 ± 8.2% [6.8 ± 8.1 mg], *d* = 0.9, P = 0.76; Fig 1C) following immobilization compared with their respective controls; percent change for soleus mass expressed relative to body mass was different between LRT and HRT (-9.0 ± 8.8 [-0.05 ± 0.04 mg/g], *d* = -1.0 vs. 6.2 ± 8.2% [+0.03 ± 0.03 mg/g], *d* = 0.7; P<0.05). Significant reduction in plantaris mass was observed in LRT in response to hindlimb immobilization (-18.64 ± 7.72% [-58.6 ± 24.29 mg], *d* = -2.6, P<0.01; Fig 1D), with a similar though non-significant trend observed in HRT (-8.18 ± 5.54% [-19.58 ± 13.25 mg], *d* = -1.4, P = 0.22; Fig 1D). The higher percent change in plantaris muscle loss relative to body mass in LRT compared with HRT also approached statistical significance (-16.4 ± 5.6% [-0.19 ± 0.06 mg/g], *d* = -1.5 vs. -8.5 ± 7.4% [-0.08 ± 0.07 mg/g], *d* = -1.2; P = 0.064).

Immobilization decreased muscle protein synthesis in LRT (control 5.4 ± 2.9 vs HIinLRT 1.2 ± 1.0 AU, *d* = -1.9) and HRT (control 5.6 ± 2.1 vs HIinHRT 3.4 ± 2.2 AU, *d* = -1.0, P = 0.34) in soleus muscle, however this decrease was only significant for LRT (P = 0.04; Fig 1E; S1 Raw images). In plantaris, muscle protein synthesis also decreased following hindlimb immobilization but was not different from control groups for LRT (control 9.0 ± 4.1 vs HIinLRT 4.3 ± 2.7 AU, *d* = -1.4, P = 0.19; Fig 1F; S1 Raw images) or HRT (control 8.8 ± 4.5 vs HIinHRT 6.7 ± 5.3 AU, *d* = -0.4, P = 0.70).

### Principal components analysis of gene expression data

**Fig 2:**
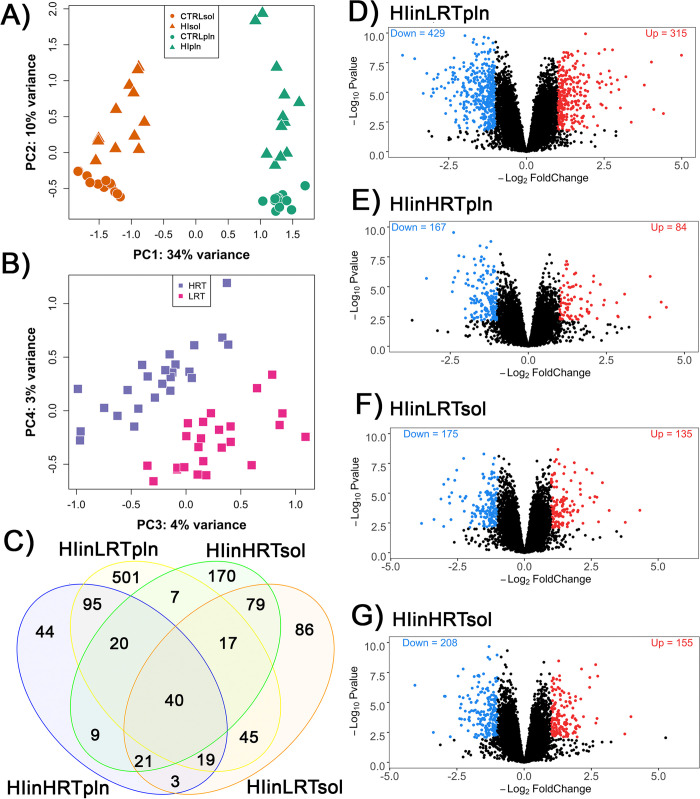
RNAseq analysis from low responders to aerobic endurance training (LRT) and high responders to aerobic endurance training (HRT) rat skeletal muscle in response to hindlimb immobilization. (**A**) Principal component analysis shows clustering of **(A)** tissue and group [PC1 vs. PC2] and **(B)** HRT/LRT line [PC3 vs. PC4]. (**C**) Venn diagram showing the overlap in differential gene expression. Volcano plots showing the 14,789 expressed genes for: (**D**) plantaris (pln) LRT hindlimb immobilization, (**E**) plantaris HRT hindlimb immobilization, (**F**) soleus (sol) LRT hindlimb immobilization, and (**G**) soleus HRT hindlimb immobilization, representing the number and magnitude of difference in expression in LRT and HRT unloaded muscle, respectively, relative to breeding line (LRT/HRT)-matched controls (logFC >1; FDR <0.05). N = 6/group.

On average, RNAseq generated 52.2 million (range = 49.6–69.8 million) reads that were mapped to 14,789 genes. There was clear variance in gene expression within and between samples groups, as demonstrated by principal component analysis (PCA; Fig 2A and 2B). PC1 showed a clear separation of plantaris and soleus muscle, illustrating tissue type had the greatest effect on gene expression variability (34%). PC2 indicated a separation of hindlimb immobilization and control groups accounting for 10% of variability in gene expression. Finally, PC3 corresponded to minor transcriptional changes based on the LRT and HRT line type (4%).

### Identification of differentially expressed genes

In total, 14,789 genes were detected in this study. Of these, there were 744 differentially expressed genes (DEGs) identified in LRT and 251 DEGs identified in HRT (logFC>1; FDR<0.05), for each plantaris hindlimb immobilization group relative to respective control groups (Fig 2D and 2E; S1 and S2 Tables in S1 File). More DEGs were downregulated than upregulated in plantaris, with 58.5% of LRT DEGs downregulated, compared with 66.5% of HRT DEGs downregulated. In soleus muscle there were 294 DEGs in LRT (57.1% downregulated) and 326 DEGs in HRT (57.1% downregulated) (Fig 2F and 2G; S3 and S4 Tables in S1 File). The expression of a subset of 40 genes was common to hindlimb immobilization in all groups and tissue types.

### Gene set enrichment analysis

**Fig 3:**
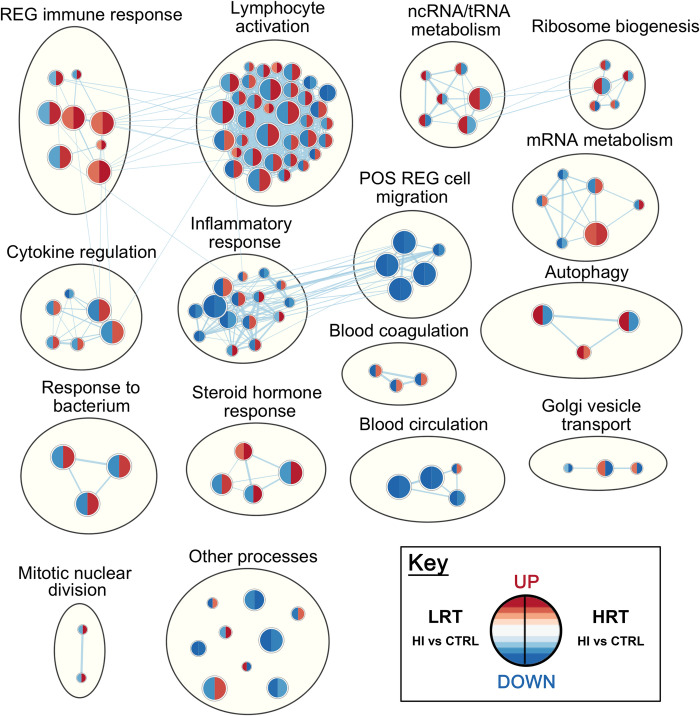
Enrichment map of Gene Ontology Biological Processes differentially expressed in low responders to aerobic endurance training (LRT) and high responders to aerobic endurance training (HRT) soleus muscle in response to hindlimb immobilization. Enrichment results were mapped as a network of gene sets (nodes) related by mutual gene overlap (edges). The enrichment map reflects relative differences (HI versus control) between LRT and HRT. Red identifies up-regulated and blue down-regulated gene sets following 3 d of hindlimb immobilization. Differential expression (LRT versus HRT) was analysed after accounting for the effect of atrophy in each group relative to their own genotypic control. The left and right side of each node indicates LRT and HRT response respectively (n = 6/group). Node size is proportional to the percent of enriched genes per set, and colour intensity represents magnitude of change in expression. Blue lines represent edges of mutual overlap. Clusters of functionally related gene sets were circled and manually labelled to highlight prevalent biological functions among a set of related gene-sets (FDR <0.05; P <0.001).

**Fig 4:**
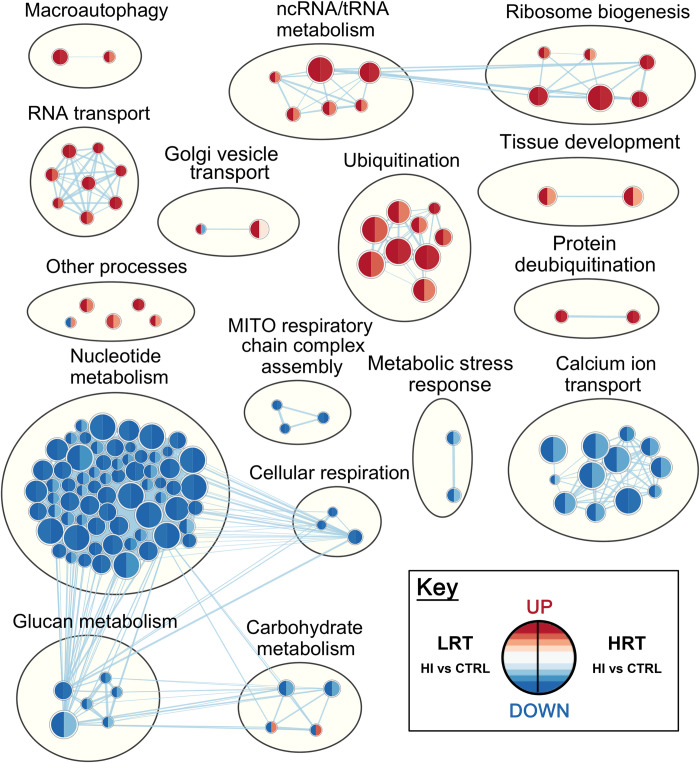
Enrichment map of Gene Ontology Biological Processes differentially expressed in low responders to aerobic endurance training (LRT) and high responders to aerobic endurance training (HRT) plantaris muscle in response to hindlimb immobilization. Enrichment results were mapped as a network of gene sets (nodes) related by mutual gene overlap (edges). The enrichment map reflects relative differences (HI versus control) between LRT and HRT. Red identifies up-regulated and blue down-regulated gene sets following 3 d of hindlimb immobilization. Differential expression (LRT versus HRT) was analysed after accounting for the effect of atrophy in each group relative to their own genotypic/phenotypic control. The left and right side of each node indicates LRT and HRT response respectively (n = 6/group). Node size is proportional to the percent of enriched genes per set, and colour intensity represents magnitude of change in expression. Blue lines represent edges of mutual overlap. Clusters of functionally related gene sets were circled and manually labelled to highlight prevalent biological functions among a set of related gene-sets (FDR <0.05; P <0.001).

**Fig 5:**
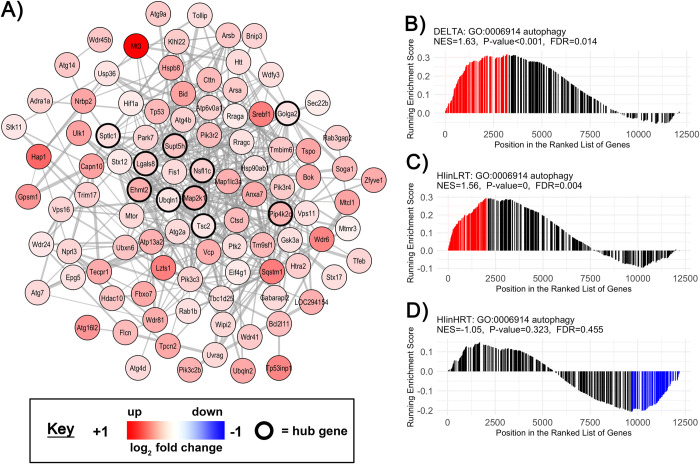
Gene set enrichment analysis of the *Autophagy* gene-set in the soleus. **A)** Co-expression network of genes involved in the *Autophagy* (GO:0006914) gene-set. Nodes correspond to individual genes significantly enriched in the delta comparison of low responders to aerobic endurance training (LRT) and high responders to aerobic endurance training (HRT; FDR<0.05, p<0.001; n = 6/group). Edge lines between two genes represent a co-expression relationship. Colour intensity represents the magnitude of dysregulation, and black borders show ‘hub’ genes in the highest 5% of connectivity within the gene-set. GSEA rank plots shown for **B)** DELTA HRT-LRT, **C)** HIinLRT and **D)** HIinHRT comparisons. On each plot the vertical lines (barcode) indicate the position of each gene within the GO:0034976 gene-set within the ranked gene list. The height of each gene is proportional to the running enrichment score. Core genes that drive the enrichment score are shown in red (positive enrichment) or blue (negative enrichment). Corresponding normalised enrichment scores (NES), p-value and FDR are also shown.

**Fig 6:**
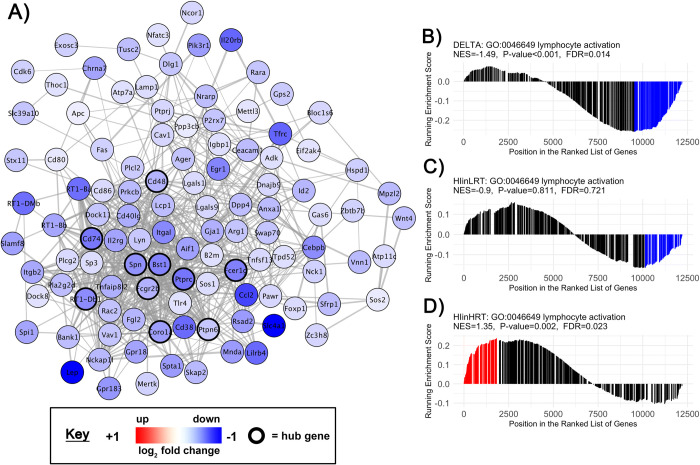
Gene set enrichment analysis of the soleus lymphocyte activation gene-set. **A)** Co-expression network of genes involved in the *Lymphocyte Activation* (GO:0046649) gene-set. Nodes correspond to individual genes significantly enriched in the delta comparison from control-to-experimental groups between low responders to aerobic endurance training (LRT) and high responders to aerobic endurance training (HRT; FDR<0.05, p<0.001; n = 6/group). Edge lines between two genes represent a co-expression relationship. Colour intensity represents the magnitude of dysregulation, and black borders show ‘hub’ genes in the highest 5% of connectivity within the gene-set. GSEA rank plots shown for **B)** DELTA HRT-LRT, **C)** HIinLRT and **D)** HIinHRT comparisons. On each plot the vertical lines (barcode) indicate the position of each gene within the GO:0046649 gene-set within the ranked gene list. The height of each gene is proportional to the running enrichment score. Core genes that drive the enrichment score are shown in red (positive enrichment) or blue (negative enrichment). Corresponding normalised enrichment scores (NES), p-value and FDR are also shown.

**Fig 7:**
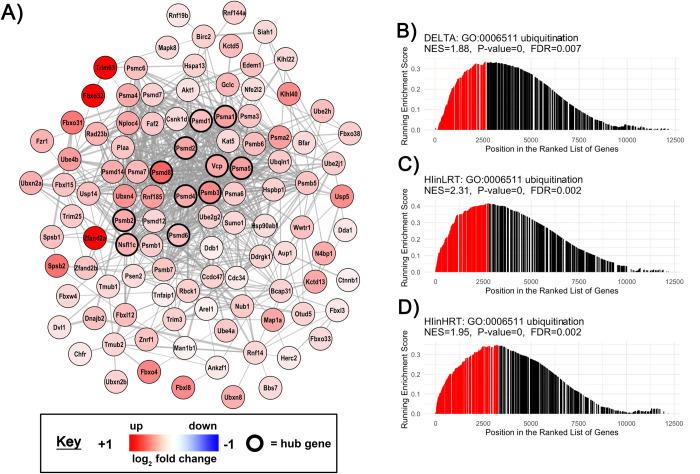
Gene set enrichment analysis of a pathway representative of the ubiquitination cluster. **A)** Co-expression network of genes involved in the *Ubiquitin-dependent protein catabolic process* (GO:0019941) gene-set. Nodes correspond to individual genes enriched in the delta comparison from control-to-experimental groups between low responders to aerobic endurance training (LRT) and high responders to aerobic endurance training (HRT; FDR<0.05, p<0.001). Edge lines between two genes represent a co-expression relationship. Colour intensity represents the magnitude of dysregulation, and black borders show ‘hub’ genes in the highest 5% of connectivity within the gene-set. GSEA rank plots shown for **B)** DELTA HRT-LRT, **C)** HIinLRT and **D)** HIinHRT comparisons. On each plot the vertical lines (barcode) indicate the position of each gene within the GO:0019941 gene-set within the ranked gene list. The height of each gene is proportional to the running enrichment score. Core genes that drive the enrichment score are shown in red (positive enrichment) or blue (negative enrichment). Corresponding normalised enrichment scores (NES), p-value and FDR are also shown.

All genes with an Entrez GeneID (12,201 genes) were ranked according to their t-statistic, then investigated by GSEA against the GO Biological Processes annotations (Figs 3 and 4; S5 and S6 Tables in S1 File). There were differences in 140 biological processes in plantaris muscle and 118 biological processes in soleus muscle when comparing LRT versus HRT after hindlimb immobilization compared to their respective control groups. In soleus muscle, 84 (of 118 total) enriched biological processes had a difference in the direction of change (i.e. positively versus negatively enriched processes) in LRT versus HRT responses to immobilization. In plantaris muscle, only 4 processes (of 140 total) were different in the direction of change. The remaining biological processes were different in the magnitude of response. Selected co-expression networks and GSEA plots illustrate dysregulation of gene-sets corresponding to specific biological processes. These are shown for *Autophagy* (GO:0006914) and *Lymphocyte activation* (GO:0046649) in soleus muscle (Figs 5 and 6), and *Ubiquitin-dependent protein catabolic process* (GO:0019941) in plantaris muscle (Fig 7). The processes selected for soleus exhibited a divergent response between LRT and HRT. The protein ubiquitination process selected for plantaris (GO:0019941) was positively enriched in both HRT and LRT in response to immobilization, however this response was greater in LRT (difference; P<0.0005).

### Expression of atrogenes and other genes of interest

**Fig 8:**
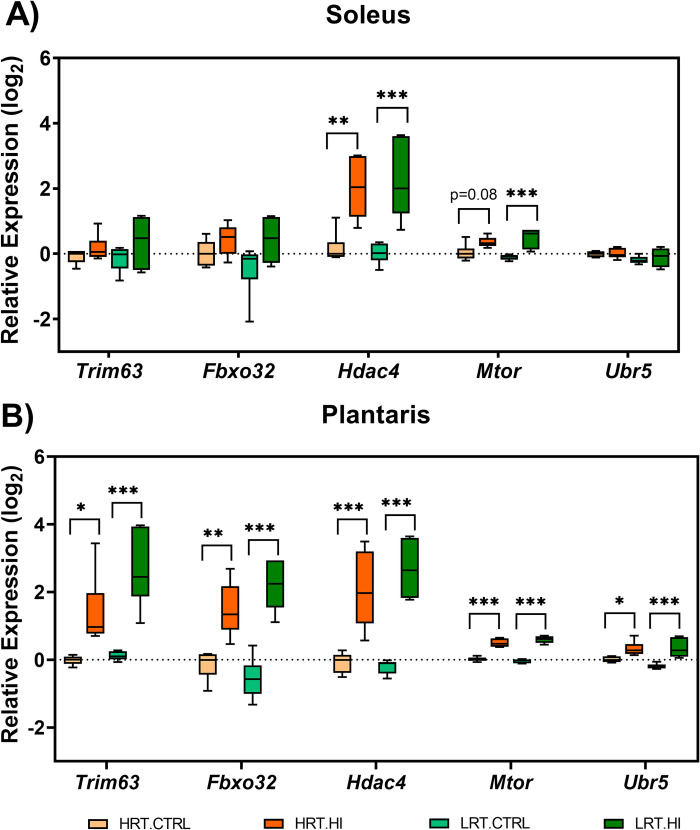
Boxplots of RNAseq data for transcripts of interest. Data is shown for *Trim63*, *Fbxo32*, *Hdac4*, *Mtor*, and *Ubr5* genes for **A)** soleus muscle and **B)** plantaris muscle in low responders to aerobic endurance training (LRT) and high responders to aerobic endurance training (HRT) control (CRTL) groups and experiment groups following 3 d hindlimb immobilization (HI). Boxes indicate the interquartile range (25%-75%) with the horizontal bar within each box indicating the median. The whiskers show the minimum and maximum values. *P<0.05, **P<0.01, and ***P<0.001 vs. line matched control (n = 6/group).

Gene expression as determined by RNAseq is shown for several genes (*Fbxo32 [Murf1]*, *Trim63 [MAFbx]*, *Ubr5*, *Hdac4 and Mtor*) with purported roles in skeletal muscle atrophy (Fig 8). In soleus muscle, only *Hdac4* and *Mtor* were upregulated and this was consistent across both HRT (*Hdac4* 1.85 log_2_fold change [FC], *Mtor* 0.32 log_2_ FC) and LRT (*Hdac4* 2.21 log_2_ FC, *Mtor* 0.59 log_2_ FC; P<0.05). In plantaris muscle *Trim63*, *Fbxo32*, *Hdac4*, *Mtor*, and *Ubr5* were also upregulated in both HRT (*Trim63* 1.41 log_2_ FC; *Fbxo32* 1.62 log_2_ FC; *Hdac4* 2.14 log_2_ FC; *Mtor* 0.49 log_2_ FC; *Ubr5* 0.32 log_2_ FC) and LRT (*Trim63* 2.55 log_2_ FC; *Fbxo32* 2.74 log_2_ FC; *Hdac4* 2.89 log_2_ FC; *Mtor* 0.65 log_2_ FC; *Ubr5* 0.54 log_2_ FC; P<0.05). There was a strong correlation between RNAseq and RT-qPCR expression of these genes (S1 Fig; S8 Table in S1 File).

Gene expression (logFC) and adjusted p-value of previously identified skeletal muscle atrogenes [24] showed differential gene expression between LRT and HRT, and soleus and plantaris muscle (S9 and S10 Tables in S1 File). Specifically, 28 atrogenes were differentially expressed in the HRT immobilization group relative to controls, compared with 33 in LRT for the soleus muscle (S9 Table in S1 File). In plantaris, 55 atrogenes were differentially expressed in HRT after three days immobilization compared with 109 in LRT (S10 Table in S1 File).

## Discussion

This study is the first to show that low responders to aerobic endurance training (LRT) have exacerbated atrophy and reduced protein synthesis compared with high responders to aerobic endurance training (HRT) following short-term immobilization. The greater atrophy in LRT soleus compared with HRT after the three-day unloading period was characterized by distinct, contrasting enrichments for gene sets in a broad range of biological processes. Differences in plantaris atrophy between LRT and HRT were less pronounced and the resulting gene-set enrichments were similarly up/down regulated but different in the magnitude of response. Together, these results provide new information on the changes in biological processes associated with the early skeletal muscle atrophy response.

Divergent gene-set enrichments with short-term atrophy between LRT and HRT were generally limited to the soleus, which is predominantly comprised of type I muscle fibres. Moreover, HRT soleus muscle appeared to be resistant to atrophy in the early (3 d) period of immobilization and maintained muscle mass comparable to HRT control animals. This apparent delay in muscle loss may be attributable to heritable genetic factors with selective breeding for high response to endurance training which may protect against short-term muscle disuse in type I fibres. Our LRT data are in close agreement with Kelleher, Pereira [25] who have previously employed the rat hindlimb casting method and report soleus muscle loss (~9%) and decreased muscle protein synthesis (~65%) following three days of immobilization. Accordingly, it appears the HRT are somewhat exceptional for their predisposition to delay the initiation of muscle atrophy with soleus muscle unloading. However, we cannot rule out the possibility that the larger soleus and plantaris muscle mass in the LRT compared with HRT control group, and variance in oedema, contributed to differences in the magnitude of atrophy, even though muscle protein synthesis was also reduced in LRT. Nonetheless, our analytical approach identifies many new gene networks contrasted between low and high responders to training that may attenuate immobilization-induced atrophy in the early phase of muscle loss.

Rapid atrophy in the early response to immobilization in soleus of LRT compared to HRT was associated with divergent enrichment of numerous biological processes, such as autophagy. Under normal homeostatic conditions autophagy is an essential process used to degrade damaged proteins and organelles via lysosomal proteolysis [26]. However, during catabolic conditions autophagy can disturb cellular protein homeostasis by increasing protein degradation [26]. The autophagy-lysosome proteolytic system degrades aggregated proteins in cells via the autophagosome and has been proposed as a mechanism mediating skeletal muscle wasting under a range of conditions including disuse [27]. Baehr, West [11] reported age-related elevations in autophagic flux in conjunction with reduced muscle fibre area in the soleus. The present data, showing differential enrichments between LRT and HRT, identifies gene networks that may underpin increased autophagy and autophagy-mediated atrophy.

The largest shift in gene-set enrichments, between HRT and LRT in soleus muscle, were for immune, cytokine regulation and lymphocyte activation biological processes. Acute immune and inflammatory processes appear to mediate muscle regeneration through several mechanisms including myogenesis [28]. It has also been proposed that changes in the transcriptome may be related to regulatory interactions between muscle, accumulated leukocytes and the extra-cellular matrix, and functionally related to inflammatory and immune responses for management of cell stress [29]. In the present study, differential enrichments for Lymphocyte Activation were particularly striking: 30 gene-sets were positively enriched in HRT and negatively enriched in LRT, representative of a coordinated response. Interestingly, cytokine-mediated recruitment of regulatory T-cells promotes regeneration [30]. In skeletal muscle, regulatory T cells express growth factors which act on muscle satellite cells, enhancing repair [30–33]. Ultimately, future work to define the immune or inflammatory response, including the time course, cell targets, and biological crosstalk may be valuable in determining how (or *if*) genetic factors attenuate atrophy via immune system related mechanisms. Undoubtedly, hindlimb cast immobilization represents a catabolic stimulus with capacity to induce cell stress and the divergent biological processes response and apparent resistance to soleus muscle loss in HRT compared to LRT in the early unloading period indicates this may be an important area for future research.

Gene sets annotated to RNA metabolism and Ribosome Biogenesis processes were upregulated in the LRT plantaris and soleus, and HRT plantaris. In contrast, HRT soleus muscle mass which was largely preserved, showed downregulation of Ribosome Biogenesis gene sets. Several studies show that disuse atrophy decreases ribosomal RNA synthesis and increases RNA breakdown, with increases in breakdown appearing to be the predominant mechanism for reductions in total RNA concentration between 7–14 d of disuse [11, 34, 35]. Given that protein synthesis is reduced after three days immobilization, the HRT soleus appears to exhibit the most appropriate response, downregulating processes associated with ribosomal biogenesis. However, further work is necessary to determine whether the HRT ribosomal response to immobilization is exceptional, with the potential to preserve muscle protein mass during unloading, and/or whether there are downstream effects on other cell processes related to the downregulation of biological pathways associated with ribosome biogenesis.

Multiple studies have reported an increase in protein ubiquitination in response to immobilization, which appears to be controlled by the atrogenes *Trim63 (Murf1)* and *Fbxo32 (MAFbx)* [36–38]. The ubiquitin-proteasome system promotes breakdown of myofibrillar proteins and has been implicated in atrophy induced by starvation, immobilization, aging, and chronic diseases [38]. Here, atrogenes *Trim63*, *Fbxo32 and Ubr5* were upregulated to a similar extent in LRT and HRT plantaris muscle with immobilization but there was a greater number of individual differentially expressed atrogenes in LRT compared with HRT in soleus and plantaris muscle. We also observed a greater upregulation of ubiquitination biological processes that were associated with more pronounced plantaris muscle loss in LRT. Interestingly, ubiquitination gene sets were not enriched in soleus muscle at the biological processes level indicating muscle fibre type may alter the time-course of the response [39, 40]. This is consistent with work by Baehr and co-workers [41] showing that at 3 days of hindlimb unloading the rate of loss in plantaris mass was twice that of soleus, before soleus loss accelerated from day 3 to day 14 [41]. Moreover, there is some evidence for a biphasic time-course for skeletal muscle atrophy where the immediate/early response to unloading is characterized by a rapid decrease in the rate of protein synthesis, followed by a subsequent increase in protein ubiquitination and proteasome activity with prolonged muscle disuse [41]. A limitation of this study is that we were unable to quantify fibre type specific cross-sectional area. In addition, data are restricted to a single time-point in the early period of the atrophy response and further work is needed to determine biological process changes at multiple time points to encompass immediate, short-term, and long-term muscle atrophy.

The largest biological process shift in plantaris muscle was a downregulation of numerous nucleotide metabolism pathways, for which there was a difference in magnitude but not direction, between LRT and HRT rats compared to their respective control groups. Nucleotides are central in regulating many cellular processes including protein metabolism, transcription/translation, intracellular signalling, and chemical energy. The more pronounced negative enrichment in nucleotide metabolism in LRT is likely related to a general impairment of metabolic processes compared with HRT [4]. For example, differences in the magnitude of negative enrichment in calcium ion transport were also evident between LRT and HRT. Given calcium flux regulates the activation of many intracellular signalling pathways and is essential for skeletal muscle contraction the greater negative enrichment in LRT also indicates metabolic impairment [42]. Moreover, there was divergent enrichment for carbohydrate metabolism processes in HRT and LRT plantaris muscle. Skeletal muscle disuse is associated with decreased glucose uptake and insulin resistance [43], and the positive enrichment in HRT compared to LRT indicates LRT have reduced capacity to maintain cell processes for muscle glucose metabolism during short-term disuse. Indeed, our findings are in agreement with the work of Lessard and co-workers [4] who have previously shown poor glucose metabolism, dysregulated molecular signalling and impaired insulin tolerance in LRT compared with HRT [4]. Thus, inferior molecular and substrate metabolism may underpin the poor response to endurance training and muscle unloading, but HRT appear to better maintain biological processes associated with cell metabolism during short-term disuse which may attenuate the severity of atrophy. Finally, we have previously reported changes in genetic profiles of LRT and HRT in response to functional overload, and both common and distinct biological process enrichment are apparent in LRT and HRT with divergent stimuli of compensatory hypertrophy in our prior work and the disuse atrophy in the present study [8]. Moreover, it appears the transcriptome response to immobilisation induced atrophy is not simply the inverse of hypertrophy, with shared and unique responses associated with a disparity in phenotypic outcome.

The dominant mechanism inducing skeletal muscle atrophy, the time-course of dysregulation of metabolic and molecular processes, and the translational relevance of data from rodents to human biology remain areas of continued scientific scrutiny [44, 45]. Our data provide support for the contribution of both reduced protein synthesis and upregulation of atrogene expression in promoting immobilization induced muscle wasting. Moreover, despite methodological and fibre type differences in analysis of the muscle transcriptome our plantaris, but not soleus, data are in partial agreement with Abadi and co-workers [46] human model of immobilization. However, neither the myosin heavy chain expression nor pronounced decrease in mitochondrial gene enrichment in human skeletal muscle is equivalent in the current rodent model.

In summary, low responders to aerobic endurance training also show an exacerbated atrophy response when directly compared with high responders to training, indicating HRT may be somewhat protected from rapid muscle loss with short-term immobilization. Characterization of soleus muscle gene expression in response to short-term atrophy identified many contrasting biological processes associated with high endurance training responders’ capacity to delay significant loss of skeletal muscle in the early unloading period. In addition, greater plantaris muscle atrophy in LRT may be attributable to variation in the magnitude of biological process enrichment during muscle unloading. Altogether, the innovative rat model employed in this study demonstrates that heritable factors that promote divergent adaptation to endurance training can have a significant impact on the regulation of biological processes associated with muscle atrophy and the extent of short-term loss of skeletal muscle.

## Supporting information

S1 Fig Pearson’s correlation x-y plot of RNA sequencing and RT-qPCR data. (TIF)

S1 Raw images Raw images for Western blotting in plantaris and soleus muscle of low responders to endurance training (LRT) and high responders to endurance training (HRT) intervention and control groups. (PDF)

S1 File Raw data for gene expression in plantaris and soleus muscle of low responders to endurance training (LRT) and high responders to endurance training (HRT) intervention and control groups. Data are shown for differentially expressed genes (S1–S4), enrichment mapping (S5, S6), primer sequences (S7), RNAseq and RT-qPCR correlation data (S8), atrogene expression (S9, S10) and myosin heavy chain expression (S11).
(XLSX)
