# Supplementary figures and images for: Effect of short-term hindlimb immobilization on skeletal muscle atrophy and the transcriptome in a low compared with high responder to endurance training model
Source: PLoS One. 2022 Jan 13;17(1):e0261723. doi: 10.1371/journal.pone.0261723 (PMC8757917; doi:10.1371/journal.pone.0261723)

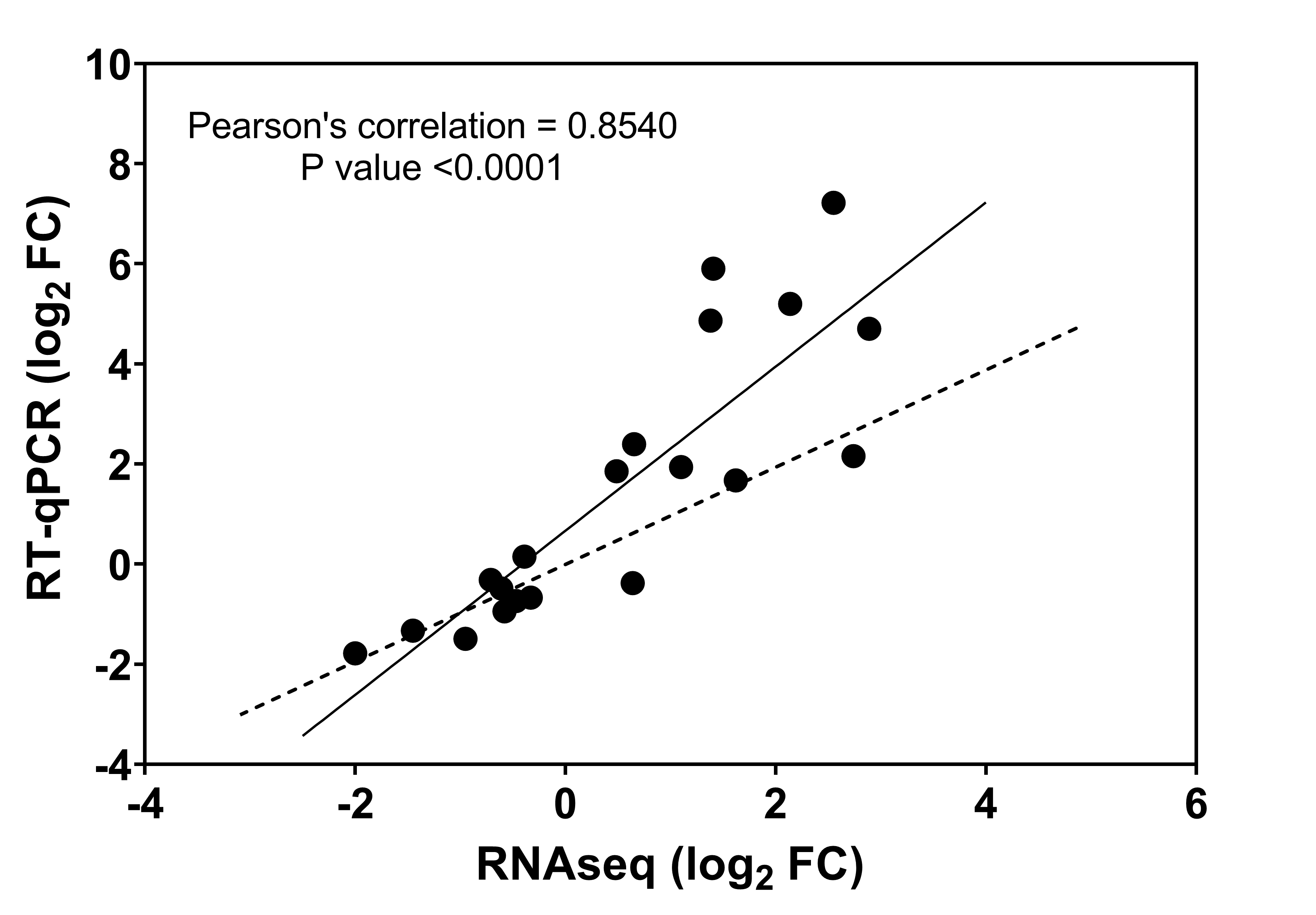

Supplement: S1 Fig — (TIF) [file pone.0261723.s001.tif]
