# Supplementary material for: Effect of short-term hindlimb immobilization on skeletal muscle atrophy and the transcriptome in a low compared with high responder to endurance training model
Source: PLoS One. 2022 Jan 13;17(1):e0261723. doi: 10.1371/journal.pone.0261723 (PMC8757917; doi:10.1371/journal.pone.0261723)
Supplement: S1 Raw images — (PDF) [file pone.0261723.s002.pdf]

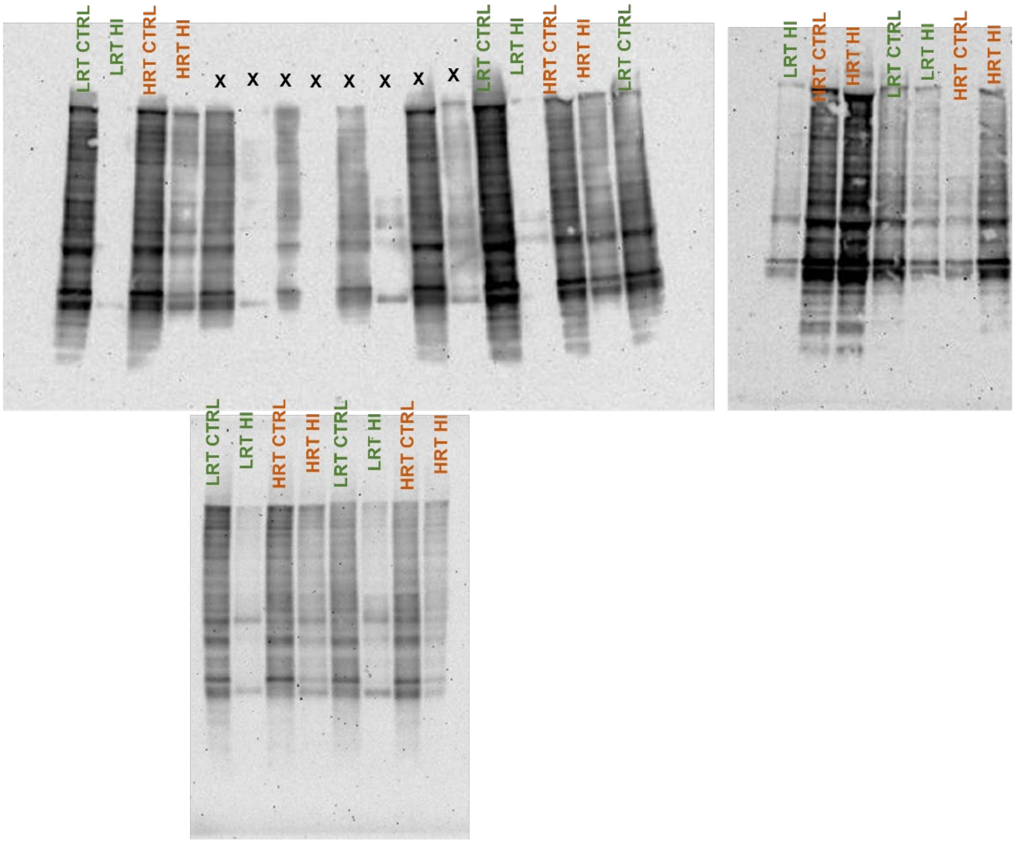

S2a. Soleus puromycin blots quantified for Figure 1E

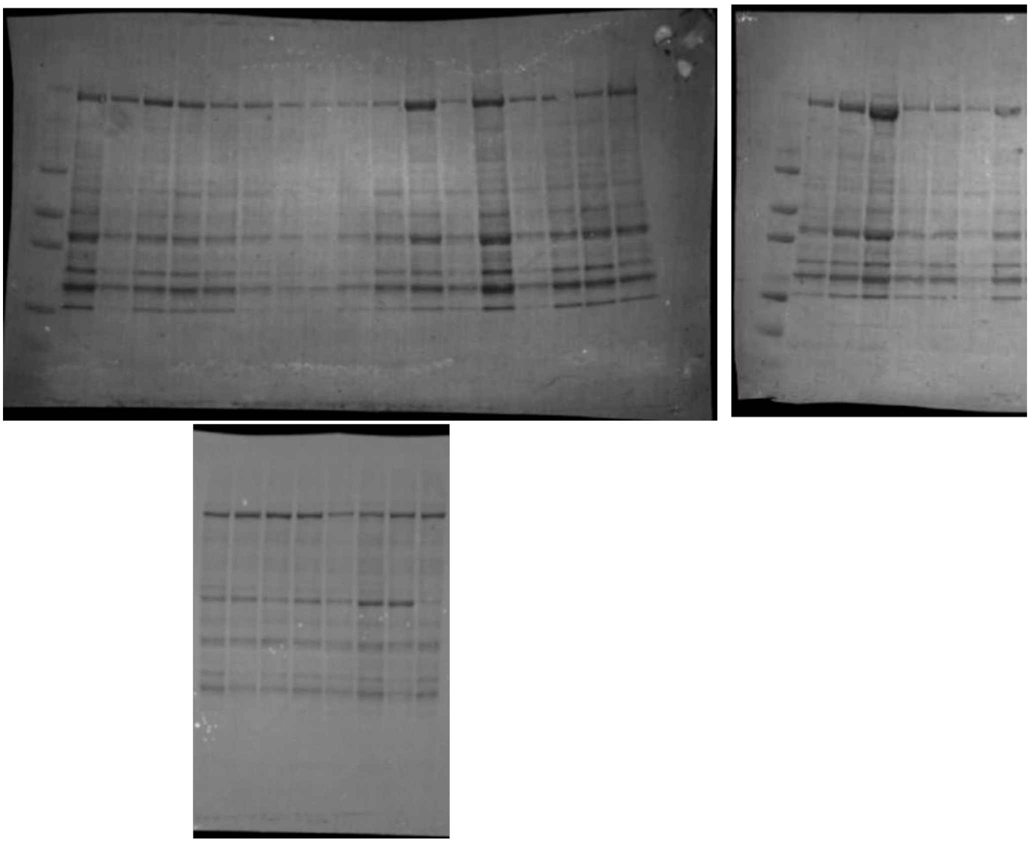

S2b. Amido black for soleus.

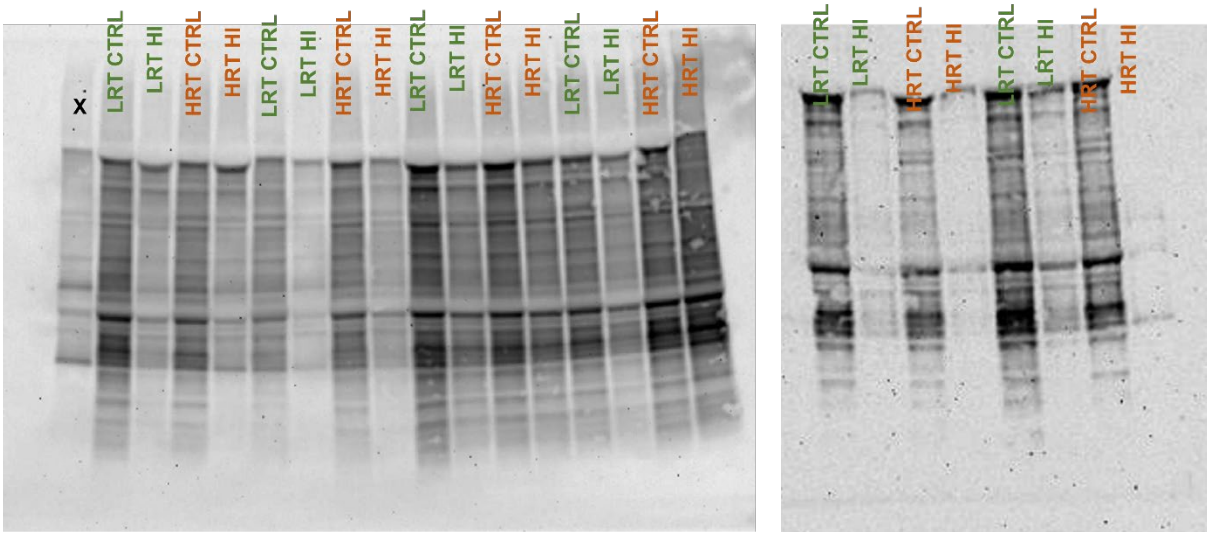

S2c. Plantaris puromycin blots quantified for Figure 1F

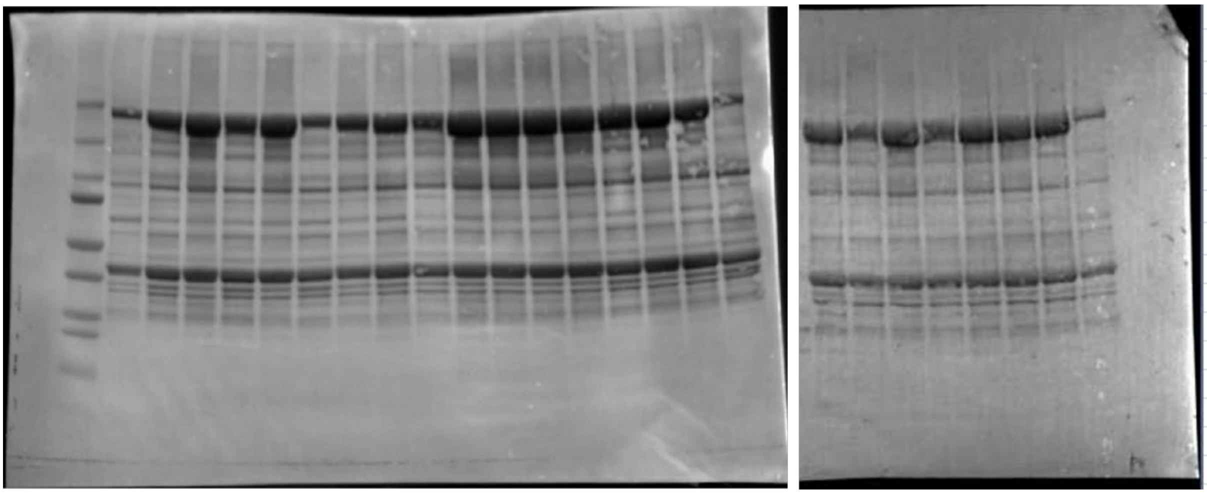

S2d. Amido Black for plantaris
